# Developmental milestones of the autonomic nervous system revealed via longitudinal monitoring of fetal heart rate variability

**DOI:** 10.1371/journal.pone.0200799

**Published:** 2018-07-17

**Authors:** Uwe Schneider, Franziska Bode, Alexander Schmidt, Samuel Nowack, Anja Rudolph, Eva-Maria Doelcker, Peter Schlattmann, Theresa Götz, Dirk Hoyer

**Affiliations:** 1 Department of Obstetrics, Division of Prenatal Diagnostics and Fetal Physiology, Jena University Hospital, Jena, Germany; 2 Hans Berger Clinic of Neurology, Biomagnetic Center, Jena University Hospital, Jena, Germany; 3 Institute of Biomedical Engineering and Informatics, Technical University, Ilmenau, Germany; 4 Institute for Medical Statistics, Computer and Data Sciences, Jena University Hospital, Jena, Germany; University of Adelaide, AUSTRALIA

## Abstract

**Background:**

Fetal heart rate variability (fHRV) of normal-to-normal (NN) beat intervals provides high-temporal resolution access to assess the functioning of the autonomic nervous system (ANS).

**Aim:**

To determine critical periods of fetal autonomic maturation. The developmental pace is hypothesized to change with gestational age (GA).

**Study design:**

Prospective longitudinal observational study.

**Subjects:**

60 healthy singleton fetuses were followed up by fetal magnetocardiographic heart rate monitoring 4–11 times (median 6) during the second half of gestation.

**Outcome measure:**

FHRV parameters, accounting for differential aspects of the ANS, were studied applying linear mixed models over four predefined pregnancy segments of interest (SoI: <27; 27+0–31+0; 31+1–35+0; >35+1 weeks GA). Periods of fetal active sleep and quiescence were accounted for separately.

**Results:**

Skewness of the NN interval distribution VLF/LF band power ratio and complexity describe a saturation function throughout the period of interest. A decreasing LF/HF ratio and an increase in pNN5 indicate a concurrent shift in sympathovagal balance. Fluctuation amplitude and parameters of short-term variability (RMSSD, HF band) mark a second acceleration towards term. In contrast, fetal quiescence is characterized by sequential, but low-margin transformations; ascending overall variability followed by an increase of complexity and superseded by fluctuation amplitude.

**Conclusions:**

An increase in sympathetic activation, connected with by a higher ability of parasympathetic modulation and baseline stabilization, is reached during the transition from the late 2nd into the early 3rd trimester. Pattern characteristics indicating fetal well-being saturate at 35 weeks GA. Pronounced fetal breathing efforts near-term mirror in fHRV as respiratory sinus arrhythmia.

## Introduction

Developing the autonomic ability to adapt to varying amounts of supply and demand in the organism is one of the key necessities during fetal maturation. This process of autonomic regulation in utero reaches a pace that may never be observed again later in life. With regard to the concept of ‘developmental origins of adult disease’, maturation in utero may be highly susceptible to acute and chronic influences with long-lasting consequences [1,2].

Autonomic capacity is mirrored in cardiovascular regulation. Since the autonomic nervous system (ANS) is involved in the regulation of nearly all organs, cardiac autonomic control, which is accessible from fetal heart rate patterns (fHRP), provides relevant diagnostic and prognostic information, not only on current fetal well-being, but also on functional autonomic brain age [3,4].

On the basis of neuroanatomical studies, a sequential development of the ANS has been suggested [5]: Both the differentiation of the lateral zone of the hypothalamus, as well as an increasing myelination of the vagal nerve, indicate a first propelling maturation of the parasympathetic branch at the end of the second trimester [6,7]. From 32 weeks of gestation (WGA) onwards, both baroreflex responsiveness and the observation of increasing respiratory sinus arrhythmia indicate advancing modulatory capacities of the vagal nerve. Simultaneously, the coordination of fetal movements and requirement driven heart rate accelerations can be seen as the expression of advancing sympathetic responsiveness [8]. Therefore, different essential periods of ANS developmental dynamics such as (a) the transitional period from the late second into the early third trimester, (b) the time around 30 to 32 WGA, and (c) the near-term pregnancy beyond 35 WGA, can be expected.

HRV analysis is nowadays an internationally standardized procedure to assess the autonomic regulation of the cardiovascular system [9]. It involves the extraction of linear and non-linear (complexity) parameters the train of cardiac beat-to-beat cycles. Interpretation requires *a priori* knowledge of the physiological background of the heart beat sequences Linear analyses have in previous studies been proven viable in the fetus, both in time and frequency domains.) [9–13]. The temporal resolution of the cardiac inter-beat intervals considerably influences the results. Therefore, an electrophysiological method of recording, i.e. fetal magnetocardiography (fMCG), bears methodical advantages [10]. Recently, HRV indices that reflect general principles of maturation, adaptation and self-organization, such as increasing fluctuation amplitude, increasing complexity and the formation of characteristic patterns, have been investigated. [4,14, 15].

In addition to the physiological changes with increasing WGA, the concurrent fetal neuro-behavioral state (state of fetal activity) must be taken into account when interpreting fHRP. Rest/activity cycles have been described from about 23 WGA onwards with progressing discernibility between 26 and 32 WGA. Synchronization of neuro-behavioral variables like fHRP, body movements and eye-movements are markers of the developmental integrity of the fetus [15–17]. From 32 WGA onwards state synchronization was observed in up to 80% of observation times.

Indices of both sympathetic activation and vagal modulation increase with growing fetal age [4,18–20]. The transitional period between the second and the third trimesters of pregnancy is characterized by the appearance of physiological decelerations, a steeply enhancing vagal regulation, followed by an increase in overall variability and complexity [10,21,22]. Beyond 30 weeks GA development is characterized by a widespread distribution of inter-individual characteristics of fHRV. This distribution can in part be related to the concurrent fetal neurobehavioral states [18]. There is a strong interconnection between fHR accelerations and intentional body movements [23].

Therefore, several gestational segments of different maturational pace are proposed: a developmental surge of the sympathetic nervous system from the late second into the early third trimester, followed by a transitional period around 30 WGA [21]. The late third trimester is characterized by (1) an increase in heart rate patterns indicative of respiratory sinus arrhythmia in association with fetal thoracic movements and (2) the synchronization of accelerative fHRP in association with fetal activity. A longitudinal study overcoming the inter-individual variance and taking these different stages of maturation into account is pending. The aim of the present study is therefore, to augment the evidence of these critical periods of normal autonomic development in utero from fHRV analysis.

## Materials and methods

### Study population

This is a prospective longitudinal observational study in a cohort of healthy pregnant women and their singleton fetuses, with normal course of pregnancies according to standard maternity care in Germany and normal maternal and perinatal outcomes. All subjects voluntarily participated and gave their written consent after explicit information on the character of the investigation. The study was approved by the local Ethics Committee of the Medical Faculty of the Friedrich Schiller University of Jena (Reg. Nr 1104-04/03 and amendments).

From the initially recruited 61 subjects one had to be excluded retrospectively because of a prenatally unknown congenital heart defect. All the 60 neonates were reported to be healthy and mature at birth. As *a priori* exclusion criteria we considered: maternal age < 18 y, multiplets, active preterm labor, cardiovascular disease, administration of any medication with known cardiovascular effects, diseases from the gestational hypertensive spectrum, maternal diabetes, substance abuse (nicotine, alcohol, drugs), hints of fetal distress from conventional fetal monitoring (CTG, ultrasound), intrauterine growth restriction/abnormal findings in Doppler ultrasound, known chromosomal or other congenital abnormalities, as well as previous exposure to synthetic steroids to enhance fetal lung maturation.

312 monitoring sessions in non-stress situations from 60 fetuses between 19 and 39 completed WGA were included in our analysis. Initially, a biweekly pattern of follow-up was intended. The numbers of repetitive sessions ranged from 4–11 over the second half of pregnancy (mean 6 sessions/subject).

Therefore, on the basis of our presuppositions, the gestational period of interest was subdivided into four developmental segments of interest **(SoI {1–4}):** <27 WGA; 27+0–31+0 WGA; 31+1–35+0 WGA; >35+1 WGA, constituting respective developmental milestones of interest. This resulted in at least one monitoring session in each of the 4 SoI per subject.

### Methods

All magnetocardiographic (fMCG) monitoring sessions were performed over 30 min, sampled at 1024 Hz in a magnetically shielded room at the Biomagnetic Center, Department of Neurology, Jena University Hospital using the vector-magnetograph ARGOS 200 (ATB, Chieti, Italy) during normal daytime working hours and according to published standards of procedure [4,24–26]. The pregnant women were positioned supine or with a slight twist to either side to prevent compression of the inferior vena cava by the pregnant uterus. The Dewar containing the magnetometers was positioned with its curvature above the fetal heart after sonographic localization and as close to the maternal abdominal wall as possible without direct contact [27]. A maternal 3-lead-ECG was additionally used to distinguish between fetal and maternal cardiac representation in the multichannel magnetic signal in cases of equivocalness.

After removal of maternal cardiac signals by independent component analysis [23], the fetal heartbeats were automatically detected and normal-to-normal (NN) beat intervals series were calculated. The NN series were screened for artifacts, arrhythmias and nonstationarities both automatically and visually. The rate of interpolated artifacts was below 5% in all cases [9]. The following distribution of recordings per pregnancy SoI served for further analysis: SoI {1} 84; SoI {2} 82; SoI {3} 80; SoI {4} 66.

Fetal heart rate variability analysis (fHRV) was performed using the parameters as listed in Table 1 [4,9,13,14,18,22]. Frequency analysis was based on the frequency bands as described by David et al. [13]. Parameters were grouped with regard to their representation of developmental and regulatory aspects of the ANS (see Table 1).

**Table 1:** Parameters of fetal heart rate variability.

Parameter	Calculation	Interpretation
Fluctuation amplitude and sympathetic activation		
SDNN (ms)	Standard deviation of NN intervals	Standard parameter of overall fHRV, influenced by both branches, pronounced by sympathetic activation
ACTAMP20 (ms)	20–95 inter-quantile distance of detrended NN interval series	fluctuation range of heart beat intervals
VLF (ms^2^)	Spectral power in the fetal very low frequency band (0.02–0.08 Hz)	representing baseline fluctuation
LF (ms^2^)	Spectral power in the fetal low frequency band (0.08–0.2 Hz)	fluctuation with intermediate oscillation frequencies
Parasympathetic (vagal) modulation		
RMSSD (ms)	Root mean square of the successive differences of NN intervals	Standard parameter of short term variability, primarily vagal influence
pNN5 (%)	Percentage of differences between adjacent NN intervals that are > 5 ms.	Formation of vagal rhythms
HF (ms^2^)	Spectral power in the fetal high frequency band (0.4–1.7 Hz)	Representing vagal influence and respiratory sinus arrhythmia
Pattern formation		
Skewness (a.u.)	Skewness of NN interval series	Change from predominant decelerations towards predominant acceleration patterns
Sympathovagal Balance		
VLF/LF	Ratio: very low and low frequency band power	Baseline fluctuation in relation to sympathovagal modulations
VLF/HF	Ratio: very low and high frequency band power	Baseline fluctuation in relation to vagal modulation
LF/HF	Ratio: low and high frequency band power	Sympathovagal balance
Increasing complexity		
gMSE(3) (bit_norm_)	Generalized Mutual Information at coarse graining level 3 of NN interval series	Complexity of sympathovagal modulations

List of parameters for fetal heart rate variability analysis [4,9,13].

In addition to the analysis of the entire 30 min recordings (unclassified data), 10 min sections of active and quiet fetal neurobehavioral states (quiescence/1F and active sleep/2F according to [15]) were selected from visual inspection of the heart rate pattern printout after a consensus decision by three independent obstetricians (Table 2). Episodes of active sleep could be found in 263 of the 312 recorded datasets (84.3%) and fetal quiescence of at least 10 min length in 65 traces (20.8%; SoI{1} 20, SoI{2} 15, SoI{3} 13, SoI{4} 17 recordings). Generally, a single episode per state and dataset was considered. This standardized procedure was performed blinded to the heart rate analysis, according to the standard criteria previously described and extended to gestational ages prior to 32 WGA [4,15,18,20]. Episodes of active awakeness were not considered separately due to their rare occurrence.

**Table 2:** Fetal state of activity.

Gestational Age	Fetal Quiescence minimum 10 min duration	Fetal Active Sleep minimum 10 min duration
19–31 WGA	Stable heart rate (variation of visually determined floating baseline < 10 bpm/3min) with small oscillation bandwidth (< +/- 5 bpm from floating baseline fHR) and isolated accelerations (>15 bpm over > 15 sec); floating baseline fHR does not exceed 160 bpm	Unstable fetal heart rate with variant floating baseline fHR not exceeding 160 bpm, with oscillations > +/- 5bpm, accelerations (if any) may exceed 160 bpm
32–41 WGA		Variant fetal heart rate with oscillation bandwidth exceeding +/- 5 bpm from floating baseline, frequent accelerations (>15 bpm, >15 sec), fHR exceeding 160 bpm only during accelerations
Reference to Nijhuis et al., 1982	Fetal Heart rate pattern A heart rate stable, with a small oscillation bandwidth. Isolated accelerations occur. These are strictly related to movements.	Fetal Heart rate pattern B Heart rate with a wider oscillation bandwidth than fHRP A and frequent accelerations during movements.

Characterization of fetal neuro-behavioral states from fetal heart rate patterns [15–18]

The original 312 recordings (RR interval series over 30 min) and the supporting information file, including the recording IDs and gestational age at each recording, constitute the minimal data set and will be made available by a download directory of the Biomagnetic Center, Department of Neurology, Jena University Hospital (please, contact praenataldiagnostik@med.uni-jena.de using the keyword biomagnetic data for details)

### Statistical analysis

Our described longitudinal design of non-independent recordings at different time points and with inconsistent representation of the single subjects required the development of a special statistical model that sufficiently takes all these aspects into consideration [28]. We therefore applied a linear mixed model; the so-called PRO MIXED (SAS 3.4. Basic Edition) [29,30]. The PRO MIXED model offers the opportunity to perform both the restricted maximum likelihood (REML) and the maximum likelihood methods to account for inter- and intra-correlation between the datasets. In the described investigation the REML method was used for the smaller estimation error and combined with the between-within-method to calculate the degrees of freedom in non-independent data sets from one subject [29]. Within the 4 separate SoI the ‘Estimator’, as a measure of developmental dynamics, was calculated for each parameter from both the state independent (30 min) and pre-selected state-dependent NN interval segments (10 min). To account for differences between the predefined SoI the linear ‘Contrast’ was applied. The Quality of the model was confirmed by zero-model likelihood ratio, significance was tested by χ^2^ test (p<0.05, trend p<0.10).

## Results

**Fig 1:**
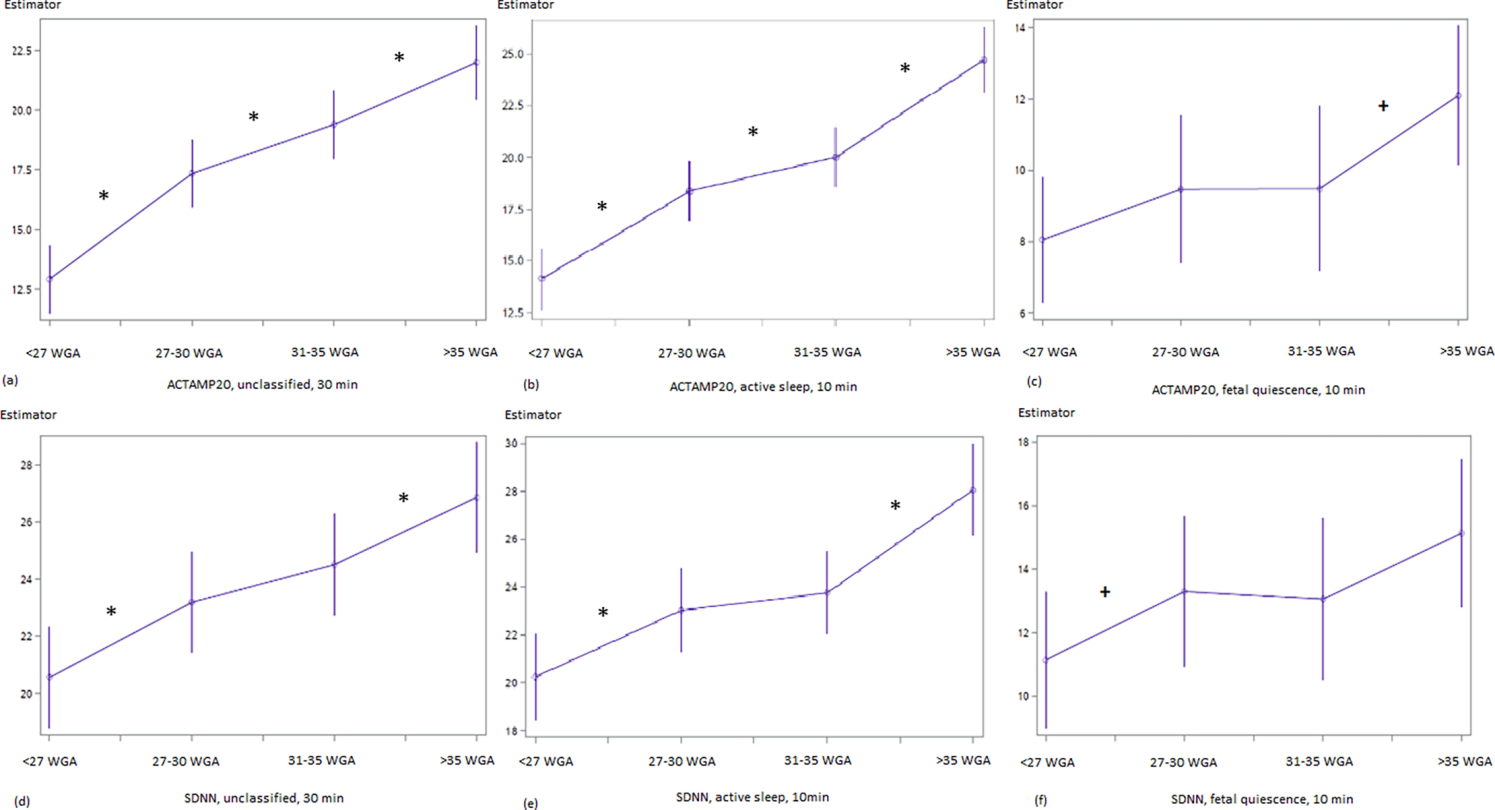
**Developmental course of ACTAMP20 (a-c) and SDNN (d-f) in the linear mixed model.** (a, d) unclassified datasets, (b, e) fetal active sleep, (c, f) fetal quiescence. x–Segment of Interest {< 27 WGA; 27–30 WGA; 31–35 WGA; >35 WGA}; completed weeks of GA; y–Estimator in [ms] units. Numerical data see Table 2. Significant (p<0.05) contrasts are marked by *, trends (p<0.10) by +.

**Fig 2:**
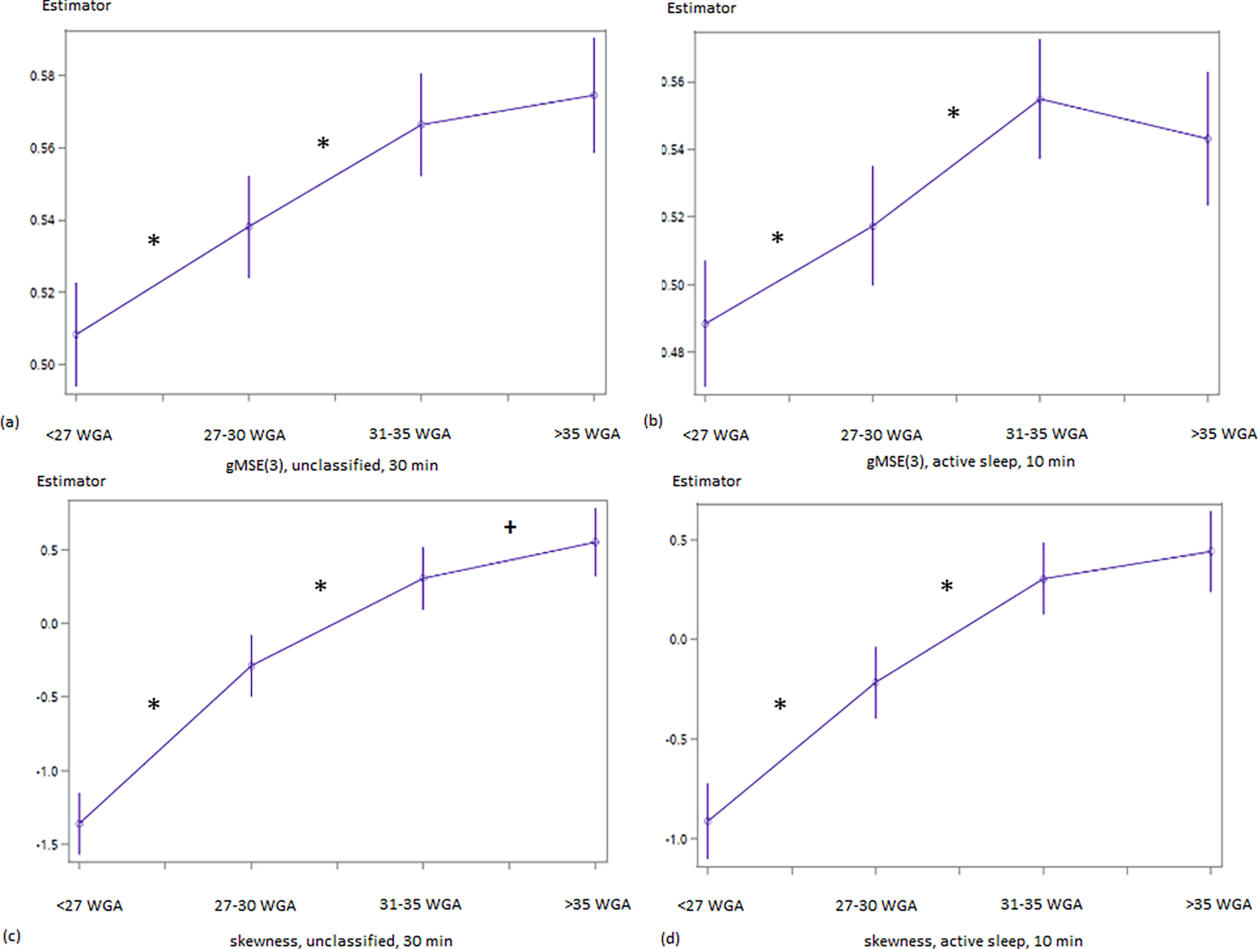
**Developmental course of gMSE(3) (a, b) and skewness (c, d) in the linear mixed model.** (a) unclassified datasets: SoI {1–2} χ^2^ = 11,81; p<0.001, SoI {2–3} χ^2^ = 10.04; p = 0.0013; (b) fetal active sleep SoI {1–2} χ^2^ = 6.7, p = 0.0096, SoI {2–3} χ^2^ = 12.17, p<0.001; (c) unclassified datasets: SoI {1–2} χ^2^ = 70.31, p<0.0001, SoI {2–3} χ^2^ = 21.29, p<0.001, SoI {3–4} χ^2^ = 3.16, p = 0.076; (d) fetal active sleep: SoI {1–2} χ^2^ = 35.50, p<0.0001, SoI {2–3} χ^2^ = 21.13, p<0.0001. x–Segment of Interest {< 27 WGA; 27–30 WGA; 31–35 WGA;>35 WGA}; completed weeks of GA; y–Estimator in [bit_norm_] Significant (p<0.05) contrasts are marked by *, trends (p<0.10) by +. Results for ‘fetal quiescence’ are not shown because they did not reach significant levels (gMSE(3): SoI {2–3}, χ^2^ = 3.41; p = 0.07).

The parameters indicating fluctuation amplitude/sympathetic activation, pattern formation and complexity were consistent, when comparing the results from unclassified 30-min-recordings and those segments representing active fetal sleep (Figs 1 and 2). An overview of the numerical data is given in Table 3.

**Fig 3:**
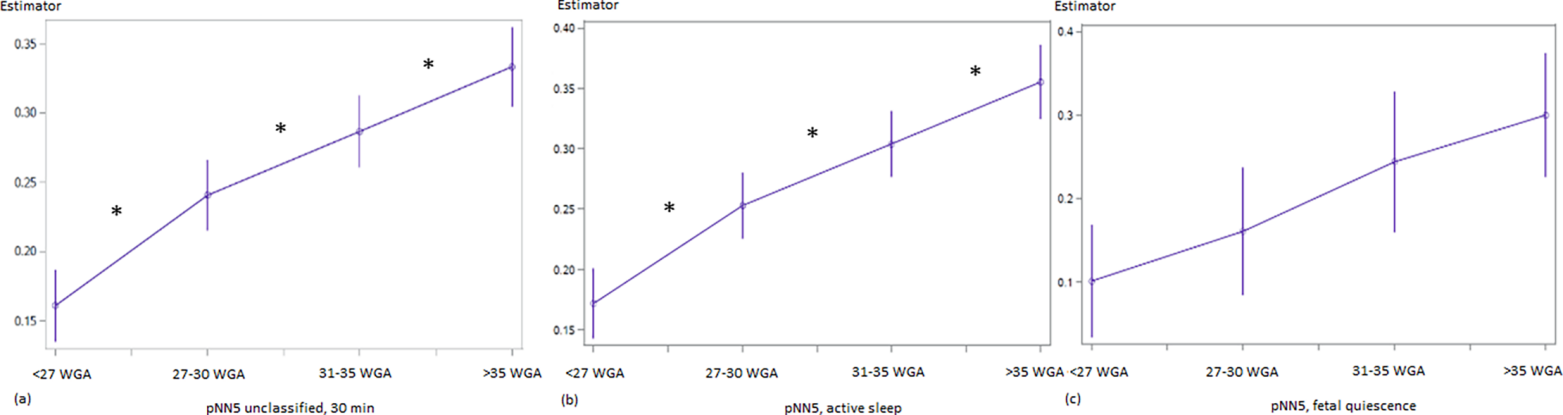
Developmental course of pNN5 in the linear mixed model. (a) unclassified datasets, (b) fetal active sleep, (c) fetal quiescence. x–Segment of Interest {< 27 WGA; 27–30 WGA; 31–35 WGA; >35 WGA}; completed weeks of GA; y–Estimator in [%]. Numerical data see Table 2. Significant (p<0.05) contrasts are marked by *, trends (p<0.10) by +.

ACTAMP20 and SDNN display a two-step-increase that is pronounced between SoI {1–2} and SoI {3–4} (Fig 1, Table 3). The observed increase in overall fluctuation amplitude is flanked by a steady increment of short term variability throughout the gestational period of interest (pNN5, Fig 3, Table 3). In addition, RMSSD (unclassified χ^2^ = 3.59, p = 0.06; active sleep χ^2^ = 7.82; p = 0.005) and HF (Table 3) show their most remarkable increase between SoI {3–4}. In contrast, both skewness and complexity follow a saturation function towards term. VLF/LF (unclassified, increase, p = 0.0098) and LF/HF (active sleep, decrease, p = 0.0624) ratios changed between SoI {1–2} (Table 2).

**Table 3:** Contrast analysis of fHRV parameters between pregnancy segments of interest.

fHRV Parameter	SoI {1} vs SoI {2}		SoI {2} vs SoI {3}		SoI {3} vs SoI {4}	
	χ^2^	Pr > χ^2^	χ^2^	Pr > χ^2^	χ^2^	Pr > χ^2^
**30 min**						
**ACTAMP20**	**34.72**	**<0.0001**	**7.42**	**0.0065**	**10.23**	**0.0014**
**SDNN**	**7.37**	**0.0066**	1.83	0.17	**5.04**	**0.025**
**pNN5**	**26.89**	**<0.001**	**8.87**	**0.0029**	**7.90**	**0.005**
**VLF**	0.03	0.8585	0.08	0.7726	1.73	0.1881
**LF**	*3*.*16*	*0*.*0754*	0.73	0.3940	0.51	0.4772
**HF**	0.85	0.3567	0.28	0.5978	**6.57**	**0.0104**
**LF/HF**	*2*.*70*	*0*.*1000*	0.40	0.5271	2.15	0.1423
**VLF/HF**	0.03	0.8705	0.11	0.7404	1.09	0.2963
**VLF/LF**	**6.67**	**0.0098**	1.04	0.3082	0.07	0.7957
**active sleep**						
**ACTAMP20**	**26.23**	**<0.001**	**4.15**	**0.0416**	**29.73**	**<0.001**
**SDNN**	**7.45**	**0.0063**	0.54	0.4615	**16.40**	**<0.001**
**pNN5**	**20.9**	**<0.001**	**8.88**	**0.0029**	**7.74**	**0.0054**
**VLF**	0.15	0.7002	0.03	0.8592	2.35	0.1249
**LF**	0.90	0.3437	0.55	0.4592	1.27	0.2602
**HF**	2.63	0.1052	0.33	0.5662	**6.65**	**0.0099**
**LF/HF**	*3*.*47*	*0*.*0624*	0.00	0.9513	1.27	0.2595
**VLF/HF**	1.38	0.2403	0.02	0.8963	0.82	0.3665
**VLF/LF**	1.08	0.2992	0.02	0.8797	0.47	0.4938

Contrast analysis of the Estimators for selected time domain, frequency domain parameters and their ratios: VLF, LF, HF, VLF/LF, VLF/HF, LF/HF. **bold p<0.05**; *italics p<0*.*10;* SoI–Segment of Interest {1 - < 27 WGA; 2–27–30 WGA; 3–31–35 WGA; 4 –>35 WGA}; completed weeks of GA

With restriction to the low number of observed periods of **fetal quiescence**, fHRV changes between SoI could only be demonstrated in a few cases as trends: SDNN between SoI {1–2} (χ^2^ = 2.95; p = 0.09, Fig 2C) and {3–4} (χ^2^ = 2.18; p = 0.14; Fig 2C), ACTAMP20 between SoI {3–4} (χ^2^ = 3.14; p = 0.08; Fig 2F), VLF between SoI {1–2} (χ^2^ = 5.07; p = 0.024) and gMSE(3) between SoI {2–3} (χ^2^ = 3.41; p = 0.07). The increase in pNN5 between SoI {2–3} (χ^2^ = 2.57, Fig 1C) did not reach an appropriate level of significance (p = 0.11).

## Discussion

**Fig 4:**
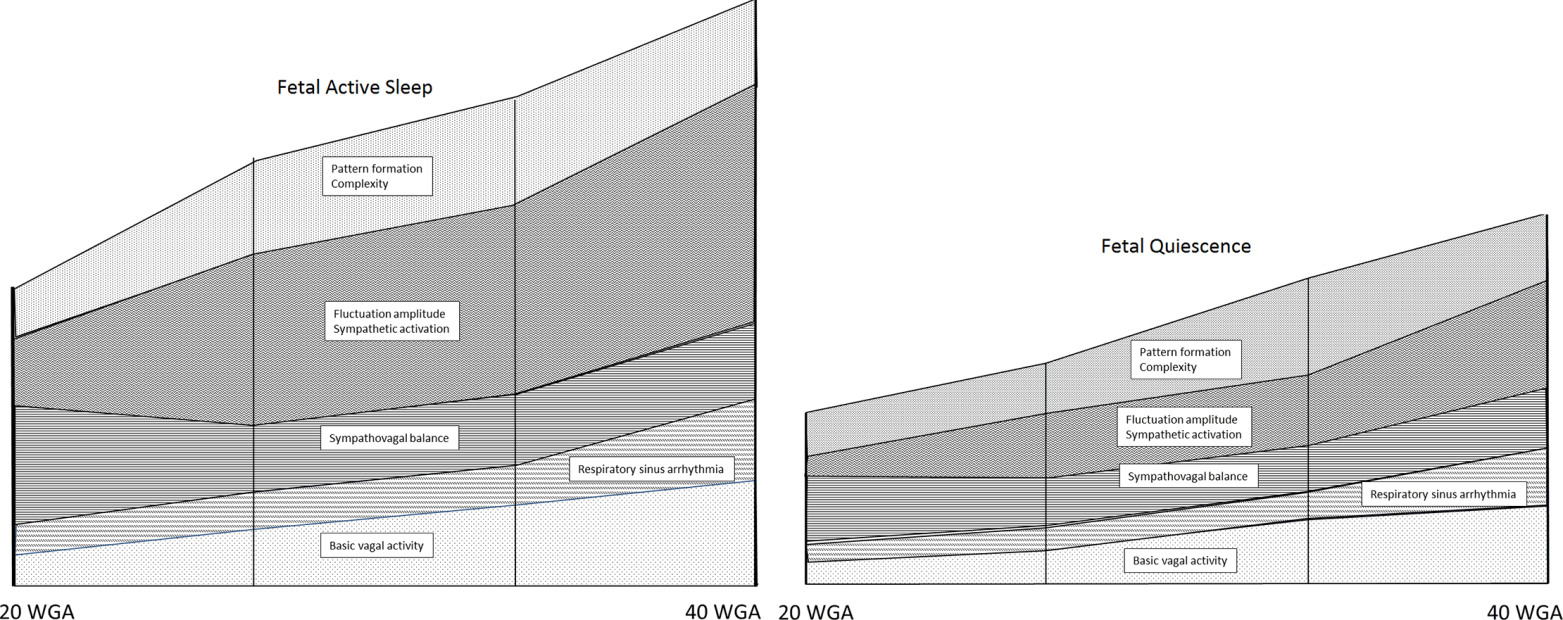
Autonomic maturation as reflected by heart rate regulation. Schematic summary of the findings for illustration purposes. The scales of the individual components contributing to heart rate regulation are arbitrary and do not reflect absolute values, but coincidental qualitative changes throughout the second half of gestation.

In this longitudinal intra-individual monitoring study we were able to demonstrate several key features of normal fetal autonomic maturation during the second half of gestation (Fig 4): The transitional period from the late second into the early third trimester is characterized by an acceleration of autonomic maturation. The general principles of maturation as described earlier are fulfilled by the increase in fluctuation amplitude (ACTAMP20, SDNN), complexity (gMSE(3)), the formation of characteristic patterns, like a shift from decelerating towards accelerative heart rate patterns (skewness), and a stabilization of baseline heart rate (VLF/LF). These characteristics are accompanied by an increase in parasympathetic modulation (pNN5, LF/HF ratio) that is more pronounced during periods of active sleep when higher sympathetic activation is self-evident (ACTAMP20, SDNN) [18]. Additionally, the period close to term is characterized by a steep increase in high frequency variability (HF, RMSSD) that most likely represents respiratory sinus arrhythmia (RSA) [31,32]. RSA is associated with breathing efforts of the maturing fetus. These thoracic movements are characteristically not confined to either of the fetal neurobehavioral states but occur more frequently during periods of active sleep [33].

Our results support the concept, that increasing sympathetic activation is accompanied by increased vagal modulation in states of preserved sympatho-vagal balance, as studied here [18]. The previous theory of ‘polyvagal development’ [5] which sees a sequential development of vagal, followed by sympathetic and again vagal maturation, can only in parts be confirmed. Our results nicely fit the two- step parasympathetic development as described from neuroanatomic studies. Cheng et al. and Porges et al. describe the maturation of primitive neurons in the parasympathetic brain stem nuclei and the development of the myelin sheaths of the vagal nerve reaching their maximum pace around 30 WGA [5,6,34,35]. In a second step, RSA is coordinated further beyond 30 WGA. Suess et al. were able to demonstrate a lack of coordination in the swallowing process to RSA in preterm infants prior to 30 WGA, in comparison to a cohort beyond 32 weeks of developmental age [36].

With focus on periods of fetal quiescence very little changes were observed during the gestational period of interest, confirming previous observations from cross-sectional studies [18,22]. Both vagal modulation (pNN5) and complexity (gMSE(3)) did solely increase around 32 WGA at the transition from SoI {2–3}. Hence, characterizing the apparent fetal state of neurobehavioral activity does essentially influence analysis of autonomic function. In previous cross-sectional studies, we were able to demonstrate an increase in beat-to-beat complexity during fetal quiescence with advancing GA [22]. Even though this is, to our knowledge, the largest prospectively conceived longitudinal study performed on fMCG heart rate monitoring so far, we finally have to contemplate, that episodes of fetal quiescence might have been underrepresented in the study population to reach significant results.

In previous work we could demonstrate, that the use of parameters like pNN5, ACTAMP20, skewness or gMSE(3) more consistently described fetal autonomic maturation than parameters primarily proposed in adult HRV (classical according to Task Force 1996) [4,9,22]. With respect to the frequency domain, a characteristic shift towards higher frequency variability from the LF to the HF spectrum has previously been described. Hence, a loss of additional information in the VLF/LF ratio from about 32 WGA onwards is to be expected [11,37].

The different HRV indices mainly address different physiological aspects. There are some known redundancies concerning vagal activity (RMSSD, pNN5, HF) and overall fluctuation range (SDNN, ACTAMP20). Therefore, we refrained from performing multiple-testing adjustments for different physiological aspects. The linear mixed model statistics inherently consider all effects and repetitions for the chosen HRV indices.

Obviously, visual analysis of the fetal neurobehavioral state of activity, solely from heart rate patterns, doesn’t represent the gold standard [15,16]. Nevertheless, the described method has been consistently used over the last years, was able to cluster fHRV results accordingly and has been adapted by research colleagues worldwide [4,14,18–22,37,38]. More recent efforts have been made to include fetal movements into analysis [23,39]. The resemblance of results between unclassified data and those, attributed to active fetal sleep in our present study, makes it unlikely that additional information on state appearance will cast doubt on the general principles elaborated here.

Continuous antenatal heart rate tracing is based on two physical principles: (i) Doppler ultrasound-based cardiotocography (CTG), although not able to identify each individual heart beat delivers a reasonable temporal resolution for daily clinical practice and algorithms for computerized analysis, i.e. according to the Dawes/Redman criteria, aid in reducing its shortcomings [40–42]. (ii) In comparison to the CTG, electrophysiological methods such as fetal electrocardiography (fECG) or fetal magnetocardiography (fMCG) allow the precise QRS complex detection.

Magnetocardiography, though sophisticated and providing optimum temporal resolution, failed to reach worldwide significance throughout its recent 40 years of application [43]. The method is far too costly and specialist-driven to be widely distributed. Moreover, fECG is burdened with severe signal attenuation due to the vernix caseosa, making it difficult to apply between 28 and 34 WGA [44–45]. Therefore, efforts should be encouraged to determine how much of the gathered information might be detectable by routine clinical applications.

## Conclusion

In conclusion, our study demonstrates changes in the developmental pace of the ANS during intrauterine life that can be monitored by selectively applying parameters of fHRV. An increase in sympathetic activation is accompanied by a higher ability of parasympathetic modulation and this maturational milestone is reached during the transitional period from the late second into the early third trimester. The near term period is characterized by pronounced periods of fetal breathing efforts that mirror in fetal heart rate regulation. The pattern characteristics of fetal active sleep are stable from 35 WGA onwards.
