# Correction: Developmental milestones of the autonomic nervous system revealed via longitudinal monitoring of fetal heart rate variability

**DOI:** 10.1371/journal.pone.0202611

**Published:** 2018-08-14

**Authors:** Uwe Schneider, Franziska Bode, Alexander Schmidt, Samuel Nowack, Anja Rudolph, Eva-Maria Dölker, Peter Schlattmann, Theresa Götz, Dirk Hoyer

The sixth author's name is spelled incorrectly. The correct name is: Eva-Maria Dölker. The correct citation is: Schneider U, Bode F, Schmidt A, Nowack S, Rudolph A, Dölker E-M, et al. (2018) Developmental milestones of the autonomic nervous system revealed via longitudinal monitoring of fetal heart rate variability. PLoS ONE 13(7): e0200799. https://doi.org/10.1371/journal.pone.0200799

## References

[1] Schneider U, Bode F, Schmidt A, Nowack S, Rudolph A, Doelcker E-M, et al (2018) Developmental milestones of the autonomic nervous system revealed via longitudinal monitoring of fetal heart rate variability. PLoS ONE 13(7): e0200799 10.1371/journal.pone.0200799 30016343 PMC6049949

